# Adapting Transportation Planning e-Tools to Older Adults’ Needs: Scoping Review

**DOI:** 10.2196/41938

**Published:** 2023-05-16

**Authors:** Sahar Tahir, Bessam Abdulrazak, Dany Baillargeon, Catherine Girard, Véronique Provencher

**Affiliations:** 1 AMbient Intelligence Lab, Département Informatique Faculty of Science University of Sherbrooke Sherbrooke, QC Canada; 2 Research Center on Aging Centre intégré universitaire de santé et de services sociaux de l'Estrie Centre hospitalier universitaire de Sherbrooke Sherbrooke, QC Canada; 3 Department of Communication University of Sherbrooke Sherbrooke, QC Canada; 4 School of Rehabilitation University of Sherbrooke Sherbrooke, QC Canada

**Keywords:** active aging, transportation, e-tool, user-centered, review, Canadian mobility experience

## Abstract

**Background:**

Aging is often accompanied by a decrease in physical and sensory capacities and financial resources, which makes travel and the use of public transport a big challenge for older adults. These mobility limitations may prevent them from going out for groceries, medical appointments, or entertainment, which increases the risk of social isolation. A key element in helping older adults to maintain healthy aging and social engagement is to foster autonomy, freedom, and active mobility. A transportation planning e-tool can provide older adults with information about transport and trip options. There are many transportation planning e-tools, but little is known about whether and how their characteristics and functionalities address older adults’ needs and preferences.

**Objective:**

This study aims to map existing transportation e-tools and identify gaps to be filled in order to match their functionalities with older adults’ needs and preferences.

**Methods:**

A scoping review of existing transportation planning e-tools was conducted based on the approach developed by Arksey and O’Malley. A search in the scientific literature (Academic Search Complete, MEDLINE, CINAHL, SocINDEX, and ERIC) as well as gray literature (TRID Database, Google Scholar, Proquest, Google Play, etc) was conducted in June 2020 and updated 3 times; in September 2021, December 2021, and May 2022. After the studies were selected, a comparative analysis was performed by 2 evaluators; an occupational therapy student and a computer science student. These e-tools were analyzed with respect to some characteristics (eg, tool’s development status, target customers, and geographic coverage) as well as 10 functionalities (time autonomy, walkability, crowd avoidance, incline avoidance, weather consideration, dark avoidance, winter obstacles avoidance, amenities inclusion, taxi driver’s information, and support affordance) that we defined based on older adults’ needs and preferences (mainly Canadians). These needs were identified from a literature review and confirmed by workshops (focus groups).

**Results:**

The scientific and gray literature search yielded 463 sources, and 42 transportation e-tools were included. None of the e-tools reviewed addresses all 10 functionalities. More specifically, functionalities such as dark avoidance and support affordance were not addressed by any of the included e-tools.

**Conclusions:**

Most of the e-tools currently available to plan trips do not address older adults’ needs and preferences. The results of this scoping review helped fill this gap by identifying functionalities to include in transportation planning e-tools designed to promote active aging. The findings of this study highlight the need to use a multicriteria optimization algorithm to address older adults’ mobility needs and preferences.

**International Registered Report Identifier (IRRID):**

RR2-10.2196/33894

## Introduction

### Background

According to the World Health Organization [1], the percentage of the population made up of older adults has grown continuously over the years. The Canadian population aged 60 years or older is estimated to be 26% of the total population in 2022 and to reach 31.2% by 2030. Aging is often associated with frailty-related difficulties, including slower walking speed and poorer balance, increasing the risk of falling on icy sidewalks or not having enough time to cross at a traffic light or climb a steep hill [2]. It is challenging for many older adults to use public transport, as they may be reluctant to get on a crowded bus or have difficulty getting on and off the bus [3]. Vision loss may prevent older adults from feeling safe when driving [4,5] while having fewer financial resources often limits the use of taxis [5,6]. As their social network is often restricted, many older adults do not have anyone to help them, for example, to go shopping [5]. In addition, a lack of digital literacy increases their difficulty planning their trips and finding public transportation options [7]. These difficulties and barriers affect older adults’ ability to move around safely and independently and prevent them from maintaining social life as actively as they would like, which is often considered essential to maintaining social connectedness, independence, and a sense of well-being [8,9]. Thus, there is a need to provide accessible and affordable transportation options and support older adults in transportation planning and selecting the means of transport that best fit their needs and preferences [10].

Transportation planning e-tools have become increasingly popular around the world, providing information about different means of transport and helping people get to their destinations quickly and easily [11]. These transportation e-tools may be helpful for basic uses, such as giving directions from a departure point to a destination or identifying the shortest and fastest route or the route with the fewest connections [11]. They can also provide real-time data (eg, bus current location and arrival time), traffic congestion, and route changes. However, little is known about the extent to which existing planning e-tools are tailored to older adults’ unique values surrounding mobility issues and whether they provide safe, independent, and pleasant trips [12,13]. Therefore, there is a need to identify gaps to be filled for e-tool functionalities to be tailored to older adults’ special needs and preferences and, by fostering their mobility, contribute to healthy aging.

### Context and Objectives

This scoping review was embedded in the first phase of a larger project named Mobilaînés. This project aims to implement a Mobility as a Service (MaaS) e-tool, or, in other words, a 1-stop platform transport service combining different means of transport and various forms of transport services to help older adults move around where, when, and how they want [14]. The Mobilaînés project is supported by LIPPA (Laboratoire d’innovations par et pour les aînés), a laboratory of innovations by and for older adults [14]. Mobilaînés is based on a living laboratory research approach, in which stakeholders from various sectors and fields collaborate to create, validate, and test new technologies, services, products, and systems in real-life contexts [14]. The ultimate aim of Mobilaînés is to promote active aging by helping older adults plan their trips and guiding them to use routes adapted to their needs (eg, avoid hills and snowy sidewalks) and preferences (pass by toilets or benches to take a rest). The aim of this scoping review was to (1) identify existing transportation e-tools designed to help with trips and that provide useful information about the various means of transportation available, (2) evaluate the extent to which their characteristics and functionalities are tailored to older adults’ needs and preferences emerging from the first phase of Mobilaînés [14], and (3) pinpoint research gaps that need to be filled in order to develop an e-tool that supports active, healthy aging.

## Methods

### Study Design

The approach used follows the five stages described by Arksey and O’Malley [15]: (1) phrasing the research questions, (2) identifying relevant libraries and sources, (3) selecting interesting transportation e-tools based on defined inclusion and exclusion criteria, (4) charting the data, and (5) summarizing the data and synthesizing the results.

### Phrasing the Research Questions

This scoping review aimed to answer the following questions:

1. What are the current local, national, and international transportation planning e-tools? What are their characteristics and functionalities?
2. To what extent do these transportation planning e-tools take older adults’ needs and preferences into consideration in order to enhance their independence, sense of well-being, and safety when moving around?

### Identifying Relevant Libraries and Sources

Relevant libraries and sources were identified by an occupational therapy student and a computer science student involved in the Mobilaînés study. The search included the scientific literature (2015–2022) in 5 databases (Academic Search Complete, MEDLINE, CINAHL, SocINDEX, and ERIC) using the following keywords: (transport* OR “public transport*” OR travel OR “public transit” OR “active transport*” OR “alternative transport” OR paratransit OR bus* OR carpool*) AND (“integrated service” OR “mobility as a service” OR “MaaS” OR “mobility information system*” OR “technology as a service” OR “TaaS” OR “intermodal mobility” OR “intermodal transportation”).

To ensure the most up-to-date review of the data, we limited our search to the scientific and gray literature (books, memoirs, and government publications) published in French or English since 2015. Our search was extended to the TRID (Transportation Research International Documentation) Database, Google Scholar, Proquest, Google, and Google Play to identify interesting mobile apps related to transport. Keywords were adapted to each source based on iterative search processes to pinpoint the most accurate and appropriate results. The keywords used for each source are shown in Table 1. Results from the databases and grey literature were exported to a reference manager (Zotero), and duplicates were eliminated.

**Table 1:** Keywords used for each source.

Source	Keywords
TRID^a^ Database	“integrated service” OR “mobility as a service” OR “MaaS” OR “mobility information system” OR “technology as a service” OR “TaaS” OR “intermodal mobility” OR “intermodal transportation”
Google Scholar	“Outil planification déplacement” (French), “Mobility as a service”
Proquest	“Mobility as a service” AND “Canada” AND (“transport” OR “public transport” OR “mobility” OR travel OR “public transit” OR “active transport” OR “alternative transport” OR “paratransit” OR “bus” OR “carpool”) AND (“integrated service” OR “mobility as a service” OR “mobility information system” OR “technology as a service” OR “intermodal mobility” OR “intermodal transportation”).
Google	“Outil planification déplacement” AND “Aide déplacement” AND “Outil aide mobilité” AND “Assistance déplacement” AND “Transport personnes âgées” (French) AND “Mobility as a service” + “for seniors” AND “Dial a ride” AND “Mobility on demand”
Google App Store or Play	“Déplacement” AND “Mobilité” (French) AND “Transport” AND “Assistive technology”

^a^TRID: Transportation Research International Documentation.

### Selecting Interesting Transportation e-Tools—Inclusion and Exclusion Criteria

Relevant sources were selected by 2 research assistants from different disciplines (occupational therapy and engineering). First, sources were screened by title and included if they (1) introduced a transportation tool or included a state-of-the-art section about transportation e-tools or (2) combined different available means of transport. Sources were then screened by abstract when available. The initial search yielded 463 sources: Academic Search Complete (n=215), MEDLINE with full text (n=30), CINAHL Plus with full text (n=14), SocINDEX with full text (n=3), ERIC (n=2), Transportation Research Board (n=104), Google Scholar (n=10), Google (n=18), Google App Store or Play (n=61), and scientific papers and conference proceedings recommended by team members (n=6). After removing duplicates, 421 publications remained, of which 379 did not meet the inclusion criteria according to title and abstract screening. Ultimately, 42 sources met the criteria defined above and were included in the full-text analysis.

### Charting the Data

The transportation planning e-tools selected were then charted in an Excel (Microsoft Corp) sheet by 2 research assistants (students in computer science and occupational therapy), according to the following characteristics: (1) transportation tool name, (2) tool’s development status, (3) tool’s interface, (4) target customers, (5) geographic coverage, and (6) cost for users. To achieve our objectives, we evaluated the selected e-tools according to a set of 7 values related to older adults’ mobility. This set of 7 values was determined by the research team (see protocol [14]) based on (1) an inventory of core values [16], and (2) older adults’ mobility needs and preferences, identified from the literature review and 6 individual phone interviews conducted during the first phase of Mobilaînés in Sherbrooke, Quebec, Canada. The following are the seven values:

1. Eco responsibility and environmental preferences: they have a great impact on older adults’ choice of means of transport. In this context, Vredin Johansson et al [17] maintained that *“…environmental preferences increase the likelihood of choosing an environmentally friendly mode over a less environmentally friendly mode.”*
2. Health: it is viewed by older adults as a state of physical, mental, spiritual, and social well-being. Daily mobility is a kind of exercise for older adults that helps them maintain an active and healthy lifestyle [14,18].
3. Safety: it is the protection of older adults’ physical, emotional, and psychological integrity. Safety concerns can create a fear of crime [19], accidents, harassment, and so on, as well as misbehavior by staff [20]. This anxiety prevents older adults from using public transportation.
4. Quality of life: a lack of transportation has a major impact on older adults’ satisfaction and sense of personal well-being. According to Kim and Ulfarsson [21], *“…mobility is significantly associated with quality of life among older people.”* Metz [18] highlighted the destination-dependent and psychological benefits of mobility.
5. Equality: it is vital to give the same consideration to promoting the mobility of all older adults. Bourgault-Brunelle [22] showed that there is a diversity of transportation services in administrative regions (of Quebec in our case), and these services are not accessible to everyone; it seems that some regions or subregions are not served as well as others. Fiedler and Consult [20] also proved that language and cultural barriers often prevent older adults from suitably using public transportation.
6. Functional autonomy: this refers to older adults’ ability to carry out their daily activities in their physical, social, institutional, and cultural environments.
7. Decision-making autonomy: this refers to older adults’ involvement in decisions that affect them. In this context, Shrestha et al [23] maintained that public transport plays a crucial role in older adults’ freedom and independence, and *“access to public transport can help older adults to avail themselves of goods, services, employment, and other activities.”*

These 7 values were then translated into 9 statements that an adapted transportation system for older adults should ideally satisfy (Table 2). To do so, 6 phone interviews with frailer older adults were conducted in order to identify facilitators and barriers to mobility (when planning trips and moving around) and document previous experiences. These phone interviews were transcribed and coded, and later on, the outcome was classified into mobility facilitators and barriers by 2 project team members and then covalidated by 2 researchers [24]. Emerging themes were compared with data from 2 workshops on mobility facilitators and barriers (see research protocol) [8]. Recurrent themes were finally analyzed by the research team to generate the Mobilaînés statements (Table 2).

A survey was sent to the steering committee members (stakeholders from the public, scientific, and community sectors who work with or study the older adult population) [8] and LIPPA’s older adults committee to validate and classify these statements according to their importance and impact on promoting active aging. Gaps between the 2 group classifications were discussed during a steering committee meeting to decide the final classification: 9 statements were classified as important, recommended, or not important. The resultant statements and the corresponding values, as well as the steering committee members’ and older adults’ classifications of these statements, are presented in Table 2.

The survey results support the relevance of co-designing a transportation tool that would offer a safe journey and routes and interfaces adapted to the physical, sensory, and cultural needs of older adults. Interactions with older adults through co-design workshops highlighted the importance of providing an easy-to-use platform and considering key elements such as the weather, especially for trips that require you to arrive at a specific time. Exploration of new travel experiences was identified as not important by both partners and the older adults committee.

To translate these statements into more technical and measurable functionalities, 2 “in-person” workshop sessions with transportation service providers and 2 others with older citizens (n=8) were conducted. In total, 10 relevant functionalities (see Table 3) were kept after the research team’s analysis and after discussion and validation by the steering committee members. These 10 functionalities are considered to be what an ideal transportation tool should provide to address older adults’ needs and preferences.

**Table 2:** Statements that a transportation tool should ideally satisfy.

Statement: Transportation tool should…	Values	Partners’ classification	Older adults’ classification
Consider the values of sustainable and eco-responsible mobility	1	Recommended	Recommended
Suggest a route adapted to older adults	3,6	Important	Important
Suggest a pleasant route	4	Important	Recommended
Be adapted and accessible to people with limitations (hearing, visual, cognitive, reading difficulties or facing linguistic or cultural barriers)	5,6	Important	Important
Foster active mobility	2,4	Recommended	Recommended
Suggest a safe journey	3,4,6	Important	Important
Be useful for more rural communities	5,6	Recommended	Recommended
Consider travel costs to support decision-making	3,5,6	Recommended	Important
Encourage the exploration of new travel experiences	4,6	Not important	Not important

**Table 3:** Relevant functionalities an ideal transportation tool should provide.

Number	Functionalities	Older adults’ interpretation
1	Time autonomy	I want to go out whenever I want (now, tomorrow, etc)
2	Walkability	I want to avoid walking or take my walking speed into account when planning trips
3	Crowd avoidance	I want to avoid crowded routes and places
4	Incline avoidance	I want to avoid hills
5	Weather consideration	I want to avoid going out when the weather is hot or wet
6	Dark avoidance	I want to avoid going out when it’s dark
7	Winter obstacle avoidance	I want to avoid icy sidewalks or icy roads
8	Amenities inclusion	I want information about relevant amenities on my route (toilet, bench, bus shelter, public telephone, etc)
9	Taxi driver’s information	I want to know who is going to pick me up, what type of car, especially for a taxi
10	Support affordance	I need some support (providing company, helping with bags and to get into, onto, and out of or off vehicles)

## Results

### Characteristics of the e-Tools

**Figure 1:**
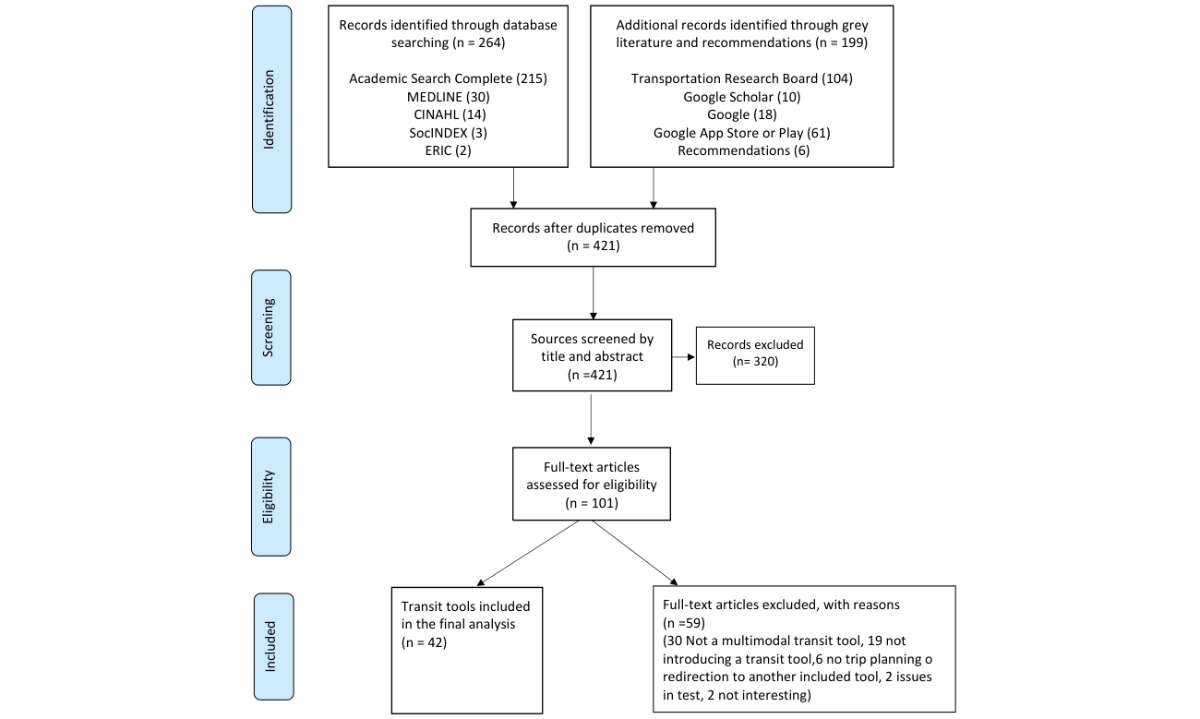
Flowchart of sources screened and included in the scoping review.

A total of 42 transportation e-tools were included in this scoping review: 37 are accessible via mobile apps (Android or iOS) and web platforms, along with 4 prototypes and 1 web platform (Figure 1). The e-tools included did not target a specific population, except for 3 of the prototypes, namely Path2.0 [25], mPASS [26], and Mobility in Later Life [27], which were designed for people with disabilities, pedestrians, and older adults, respectively. Two e-tools (STS [28] and Embarque Estrie [29]) cover the region where the study took place (the city of Sherbrooke, Quebec, Canada), while 4 e-tools cover other cities in Quebec (the Montreal area for Chrono [30], STL Synchro+ [31], and TripGo [32], the province of Quebec for Exo Quebec [33]), and 2 cover cities in another Canadian province, Ontario (OC Transpo [34] in Ottawa and Triplinx in Toronto). While 27 e-tools are for use in different cities and countries in Europe, 6 e-tools (Transit [35], Moovit [36], CityMapper [37], GoogleMaps [38], HERE WeGo [39], and Transperth [40]) can be used in different cities around the world. Most of the e-tools reviewed (n=38) consider public transport: 7 consider only bus while others consider rail, tramway, and bus. All of the e-tools reviewed suggest walking paths, 29 suggest bike paths, 8 suggest bike sharing, and 10 e-tools redirect users to ride-sharing websites or apps for ride-sharing routes. The same is true for taxi routes, which are included in 4 of the e-tools reviewed. Three e-tools redirect people to Uber for rides. Twenty e-tools suggest riding (car) paths. Five e-tools suggest paths for kick scooters, but only 1 tool suggests paths for motorbikes. All the transportation e-tools reviewed are free to install and use, except for Transit [35] and Whiz [41], which charge fees for additional personalization and more functionalities.

### Functionalities of the e-Tools

Following is our analysis of the e-tools in relation to the 10 functionalities.

- Functionality 1 (Time autonomy): all of the e-tools reviewed, except for 5 [25–27,29,42], which do not give any details about time, allow users to set a departure time and depart whenever they want. Embarque Estrie [29] does not give a choice of departure time (it shows alternate routes without further details about time), while no details were given for this functionality in the 4 prototypes examined (mPASS [26], Path2.0 [25], Sway [42], and Mobility in Later Life [27]).
- Functionality 2 (Walkability): twelve of the e-tools provide the option of minimizing distance or walking. Eleven e-tools ask users to choose their walking speed or to set the maximum walking distance tolerated, or both (walking speed and maximum walking distance tolerated). Path2.0 [25] is a prototype that stores accessible routes for people with disabilities so these routes could be recommended for the next trip; this could also be applied to walking speed.
- Functionality 3 (Crowd avoidance): five of the e-tools reviewed give information about road traffic. Three e-tools give real-time data about free parking places or an estimate of the crowd on the bus, while only one, Google Maps [38], provides an estimate of available seats in addition to estimated traffic for bus and road trips.
- Functionality 4 (Incline avoidance): inclines were not considered by most of the e-tools reviewed, except for Transp’Or [43], which suggests balanced and bike-adapted paths. Martinique mobilités [44], Irigo [45], and Tac mobilités [46] show the bike path’s elevation. Additionally, Irigo [45] and Tac mobilités [46] show the percentage of cyclable, normal, and dangerous lanes in bike paths. The prototype mPASS [26] provides personalized maps and adapted routes that consider users’ needs. For example, inclines can be considered a barrier about which data will be collected through sources (crowdsourcing, sensing, and expert data), so they can be considered when route planning. Stairs were considered in the case study of testing the prototype. Sway [42] considers the criterion of comfort when planning a route through incline avoidance.
- Functionality 5 (Weather consideration): most of the e-tools reviewed do not consider the weather, except for 4 e-tools [27,34,42,47] that give the temperature.
- Functionality 6 (Dark avoidance): this functionality was not considered by any of the e-tools reviewed.
- Functionality 7 (Winter obstacle avoidance): similar to Functionality 4, icy sidewalks were not considered by any of the e-tools reviewed, except for mPASS [26], a prototype that provides personalized maps and adapted routes that consider users’ needs. For example, icy sidewalks can be considered a barrier about which data will be collected through sources (crowdsourcing, sensing, and expert data), so they can be considered when route planning. Stairs were considered in the case study of testing the prototype. Sway [42] considers the criterion of comfort when planning a route through incline avoidance.
- Functionality 8 (Amenities inclusion): relevant amenities and services (eg, bus stations, parks, hospitals, universities, and parking) available near a given address are provided by 7 e-tools, while Embarque Estrie [29] gives relevant places near the departure and arrival addresses. Toilets and benches were not considered by any of the e-tools reviewed, except for mPASS [26], where toilets and benches can be considered facilities to take into account in route planning. Ramps and curb cuts were considered in the case study of testing the prototype.
- Functionality 9 (taxi driver’s information): none of the e-tools reviewed includes this information, except for 3 e-tools (Transit [35], OiseMobilité [48], and Go!Vermont [49]) that redirect users who choose ride-sharing to another app that gives details about the carpooler, and Mobility in Later Life [27], which shows the carpool route and details about the carpooler.
- Functionality 10 (Support affordance): none of the e-tools reviewed has the option of getting support (providing company, helping with bags, and to get into, onto, and out of or off vehicles).

Table 4 summarizes further details.

**Table 4:** Operationalization of defined functionalities by the e-tools reviewed.

Functionality and how this is addressed by the e-tools in this scoping review		Tool references
Time autonomy: provide the option of choosing a departure date and time		[27,28,30-66]
**Walkability**		
	Provide the option of minimizing distance or walking	[31,33,35,36,38,41,48,52,59,62,65,66]
	Store accessible routes for people with disabilities so these routes could be recommended for the next trip	[25]
	Consider the maximum distance tolerated by the user	[28,40,47,51,52,57,66]
	Consider the walking speed indicated by the use in route planning	[28,40,44-48,52,56,57,63,66]
**Crowd avoidance**		
	Provide information about road traffic or parking	[38,39,47,49,50,54]
	Provide an estimate of the crowd on the bus	[38,61,63]
	Provide an estimate of the crowd at the destination	[38]
**Incline avoidance**		
	Consider inclines a barrier to avoid; data about inclines could be collected through crowdsourcing, available data provided by experts	[26]
	Provide balanced and adapted routes for biking	[37,43]
	Consider the criterion of comfort	[42]
	Show the bike path’s elevation	[44-47,52,54,59]
	Show the percentage of cyclable, normal, and dangerous lanes in bike paths	[45,46,52,54]
Weather: display information about the temperature		[27,34,39,42,47]
Dark avoidance: none		None
Winter obstacle avoidance: consider icy sidewalks a barrier to avoid; data about icy sidewalks could be collected through crowdsourcing, available data provided by experts		[26]
**Amenities inclusion**		
	Have the option next to the user that gives relevant amenities (bus stations, parking, hospitals, universities, parks, administrations, etc) near the address given	[28,48,53,55,59,60]
	Show users relevant amenities (bus stations, bike stations, parking, etc) within 500 m of departure and destination locations	[29]
	Consider toilets and bench facilities to take into account; data about toilets could be collected through crowdsourcing, and available data provided by experts	[26]
Taxi driver’s information: in the case of ride-sharing, redirect users to the ride-sharing app containing further details about the carpooler, such as the person’s name and type of car		[35,48,49]
Support affordance: none		None

## Discussion

### Principal Findings

The aim of this scoping review was to explore the scientific and gray literature in order to identify existing transportation planning e-tools and evaluate the extent to which their characteristics and functionalities are tailored to older adults’ needs and preferences. Although many transportation planning e-tools have been developed to help people reach their destination using different means of transport (car, taxi, bus, car sharing, bicycle, walking, etc) and to give them various details about the trip (eg, directions, which bus to take, and which station), most of the existing e-tools focus on the functionalities for the shortest or fastest route or the route with the fewest transfers and consider only 1 criterion (distance, time, or the number of transfers). However, based on the Mobilainés project workshops, older adults did not find these functionalities (shortest or fastest route) to be very important. Furthermore, older adults’ needs with respect to avoiding winter obstacles and inclines were not taken into consideration in most of the e-tools reviewed. In general, workshop results show that weather conditions are considered by older adults when making decisions about transportation means, the time of the day, and the reason they travel. This finding is consistent with those of Stein et al [27]. Incline avoidance was considered only for bike paths by 8 e-tools. The only exceptions are mPASS [26], which considers icy sidewalks and inclines as barriers to avoid in route planning, and Sway [42], which considers the criterion of comfort. Moreover, few e-tools provide functionalities that might help older adults who have difficulty walking. Transit [35] proposes routes without stairs, while VaNavigo [56], RATP [61], and Path2.0 [25] suggest accessible routes for people with reduced mobility using wheelchairs. In this context, TripGo [32] provides 6 alternatives (recommended, greenest, easiest, fastest, healthiest, and cheapest) (see Multimedia Appendix 1 for further details).

To foster older adults’ mobility, identified gaps (eg, help to get into or onto, and out of or off vehicles and with bags or a walker), awareness of accessible amenities (eg, toilets and restaurants) as brought up by Stein et al [27], and information about whether it will be dark during the return trip) should be considered in the Mobilainés platform to fulfill their needs and preferences. Furthermore, since older adults’ needs and preferences may differ from one person to the next, multicriteria optimization algorithms may be a promising way to personalize mobility [27]. To contribute to healthy aging, there is a great need for a transportation planning tool that provides personalized maps with textual and graphic presentation and routes adapted to older adults’ physical and sensory impairments and cognitive capacities [5].

### Limitations

This study has some limitations; for example, some articles may not have been retrieved (due to the chosen keywords). Including prototypes in our scoping review is also a limitation of our analysis because these e-tools are not accessible and could not be tested. Furthermore, not including transportation planning e-tools that use only one mode may be a limitation because they may have some interesting functionalities for an e-tool involving only one mode of transport.

### Conclusions

This scoping review identified gaps that should be addressed to produce transportation planning e-tools that aim to promote active and independent aging. The results of this scoping review will be useful in designing a personalized multimodal planning tool, such as Mobilaînés, to help older adults select a route that takes their needs and preferences into account. Further research is needed to determine whether data related to the functionalities identified are available or must be created to develop a transportation planning tool in line with older adults’ values. Challenges that remain concerning include which approaches to take in terms of routing algorithms, optimization criteria, and the importance of each of the criteria considered in order to find suitable routes for older adults and make these e-tools readily accessible to users with limited digital literacy.

## Appendix

Multimedia Appendix 1 Functionalities of the reviewed e-tools.

## Abbreviations

LIPPA: Laboratoire d’innovations par et pour les aînés
MaaS: Mobility as a Service
