# Supplementary material for: Adapting Transportation Planning e-Tools to Older Adults’ Needs: Scoping Review
Source: JMIR Aging. 2023 May 16;6:e41938. doi: 10.2196/41938 (PMC10230352; doi:10.2196/41938)
Supplement: Multimedia Appendix 1 [file aging_v6i1e41938_app1.docx]

|  | **Time Autonomy** | **Walkability** | **Crowd avoidance** | **Incline avoidance** | **Weather consideration** | **Dark avoidance** | **Winter obstacle avoidance** | **Amenities inclusion** | **Taxi drivers information** | **Support affordance** |
| --- | --- | --- | --- | --- | --- | --- | --- | --- | --- | --- |
| mPASS [26] |  |  |  | Consider inclines as a barrier to avoid, data concerning inclines could be collected through crowd sourcing, available data provided by experts |  |  | Consider icy sidewalks a barrier to avoid, related data could be collected through crowdsourcing, available data provided by experts | Consider toilets and benches as facilities to take into account, corresponding data could be collected through crowd sourcing, available data provided by experts |  |  |
| Path2.0 [67] |  | store accessible routes for people with disabilities so these routes could be recommended for the next trip. |  |  |  |  |  |  |  |  |
| STS [28] | Provide the option of choosing departure date and time | Consider maximum distance tolerated and walking speed indicated by the user in route planning |  |  |  |  |  | Have the option next to the user that gives relevant amenities (bus stations, parking, hospital, universities, parks, administrations, etc.) near the address given |  |  |
| Embarque Estrie [29] |  |  |  |  |  |  |  | Show users relevant amenities (bus stations, bike stations, parking, etc.) within 500m of departure and destination locations |  |  |
| Chrono [30] | Provide the option of choosing departure date and time |  |  |  |  |  |  |  |  |  |
| OC Transpo [68] | Provide the option of choosing departure date and time |  |  |  | Display information about the temperature |  |  |  |  |  |
| STL [31] | Provide the option of choosing departure date and time | Provide the option of minimizing distance / walking |  |  |  |  |  |  |  |  |
| Exo [33] | Provide the option of choosing departure date and time | Provide the option of minimizing distance / walking |  |  |  |  |  |  |  |  |
| Transit [69] | Provide the option of choosing departure date and time | Provide the option of minimizing distance / walking |  |  |  |  |  |  | In the case of ridesharing, redirect users to the ridesharing application containing further details about the carpooler |  |
| Moovit [36] | Provide the option of choosing departure date and time | Provide the option of minimizing distance / walking |  |  |  |  |  |  |  |  |
| CityMapper [70] | Provide the option of choosing departure date and time |  |  | For bikes, it provides 3 alternatives: quiet, regular, fast |  |  |  |  |  |  |
| Google Maps [71] | Provide the option of choosing departure date and time | Provide the option of minimizing distance / walking | Provide nformation about road traffic, parking, estimate of the crowd on the bus and at the destination |  |  |  |  |  |  |  |
| HERE We Go [72] | Provide the option of choosing departure date and time |  | Provide information about road traffic |  | Display information about the temperature |  |  |  |  |  |
| Mobilite it [47] | Provide the option of choosing departure date and time | Consider the maximum distance tolerated indicated by the user and walking speed in route planning | Provide information about road traffic and parking | Show bike path’s elevation | Display information about the temperature |  |  |  |  |  |
| Oise Mobilité [48] | Provide the option of choosing departure date and time | Propose the option of minimizing distance / walking and consider walking speed indicated by the user in route planning |  |  |  |  |  | Have the option next to the user that gives relevant amenities (bus stations, parking, hospital, universities, parks, administrations, etc.) near the address given | In the case of ridesharing, redirect users to the ridesharing application containing further details about the carpooler |  |
| Go! Vermont [49] | Provide the option of choosing departure date and time |  | Provide information about road traffic and parking |  |  |  |  |  | In the case of ridesharing, redirect users to the ridesharing application containing further details about the carpooler |  |
| M-mobilités [73] | Provide the option of choosing departure date and time |  | Provide information about road traffic / parking |  |  |  |  |  |  |  |
| RTM [74] | Provide the option of choosing departure date and time | Consider the maximum distance tolerated indicated by the user |  |  |  |  |  |  |  |  |
| Le Met' [52] | Provide the option of choosing departure date and time | Propose the option of minimizing distance / walking and consider walking speed and maximum distance tolerated in route planning |  | Show bike path’s elevation and percentage of cyclable, normal and dangerous lanes in bike paths |  |  |  |  |  |  |
| Pass pass [75] | Provide the option of choosing departure date and time |  |  |  |  |  |  | Have the option next to the user that gives relevant amenities (bus stations, parking, hospital, universities, parks, administrations, etc.) near the address given |  |  |
| Modalis [54] | Provide the option of choosing departure date and time |  | Provide information about road traffic and parking | Show bike path’s elevation and percentage of cyclable, normal and dangerous lanes in bike paths |  |  |  |  |  |  |
| Divia [55] | Provide the option of choosing departure date and time |  |  |  |  |  |  | Have the option next to the user that gives relevant amenities (bus stations, parking, hospital, universities, parks, administrations, etc.) near the address given |  |  |
| ViaNavigo [56] | Provide the option of choosing departure date and time | Consider walking speed indicated by the user in route planning |  |  |  |  |  |  |  |  |
| Alterneo [76] | Provide the option of choosing departure date and time | Consider maximum distance tolerated and walking speed indicated by the user in route planning |  |  |  |  |  |  |  |  |
| Semo [58] | Provide the option of choosing departure date and time |  |  |  |  |  |  |  |  |  |
| Vrd Mobilites [59] | Provide the option of choosing departure date and time | Propose the option of minimizing distance / walking |  | Show bike path’s elevation |  |  |  | Have the option next to the user that gives relevant amenities (bus stations, parking, hospital, universities, parks, administrations, etc.) near the address given |  |  |
| Oura [77] | Provide the option of choosing departure date and time |  |  |  |  |  |  | Have the option next to the user that gives relevant amenities (bus stations, parking, hospital, universities, parks, administrations, etc.) near the address given |  |  |
| RATP [61] | Provide the option of choosing departure date and time |  | Provide estimate of the crowd on the bus |  |  |  |  |  |  |  |
| MyBus [78] | Provide the option of choosing departure date and time | Propose the option of distance / walking minimization |  |  |  |  |  |  |  |  |
| MOBIGO [79] | Provide the option of choosing departure date and time | Consider walking speed indicated by the user in route planning | Provide estimate of the crowd on the bus |  |  |  |  |  |  |  |
| GVH [64] | Provide the option of choosing departure date and time |  |  |  |  |  |  |  |  |  |
| Whim [65] | Provide the option of choosing departure date and time | Propose the option of minimizing distance / walking |  |  |  |  |  |  |  |  |
| Mobility in Later Life [80] | Provide the option of choosing departure date and time |  |  |  | Display information about the temperature |  |  |  |  | Have the option next to the user that gives relevant amenities (bus stations, parking, hospital, universities, parks, administrations, etc.) near the address given |
| Sway [81] |  |  |  | Consider the criterion of comfort | Display information about the temperature |  |  |  |  |  |
| Transp’Or [43] | Provide the option of choosing departure date and time |  |  | Provide balanced and adapted paths for biking |  |  |  |  |  |  |
| Martinique mobilités [44] | Provide the option of choosing departure date and time | Consider walking speed indicated by the user in route planning |  | Show bike path’s elevation |  |  |  |  |  |  |
| Triplinx [66] | Provide the option of choosing departure date and time | Propose the option of minimizing distance / walking and consider the maximum distance tolerated and walking speed indicated by the user in route planning |  |  |  |  |  |  |  |  |
| Whiz [41] | Provide the option of choosing departure date and time | Propose the option of minimizing distance / walking |  |  |  |  |  |  |  |  |
| Transperth [40] | Provide the option of choosing departure date and time | Consider maximum distance tolerated and walking speed indicated by the user in route planning |  |  |  |  |  |  |  |  |
| TripGo [32] | Provide the option of choosing departure date and time |  |  |  |  |  |  |  |  |  |
| Irigo [45] | Provide the option of choosing departure date and time | Consider walking speed indicated by the user in route planning |  | Show bike path’s elevation and percentage of cyclable ,normal and dangerous lanes in bike paths |  |  |  |  |  |  |
| Tac Mobilités [46] | Provide the option of choosing departure date and time | Consider the walking speed indicated by the user in route planning |  | Show elevation of bike path and percentage of cyclable, normal and dangerous lanes in bike paths |  |  |  |  |  |  |
